# A new bioindicator, shell of *Trachycardium lacunosum*, and sediment samples to monitors metals (Al, Zn, Fe, Mn, Ni, V, Co, Cr and Cu) in marine environment: The Persian Gulf as a case

**DOI:** 10.1186/s40201-016-0260-0

**Published:** 2016-10-10

**Authors:** Vahid Noroozi Karbasdehi, Sina Dobaradaran, Iraj Nabipour, Afshin Ostovar, Amir Vazirizadeh, Masoumeh Ravanipour, Shahrokh Nazmara, Mozhgan Keshtkar, Roghayeh Mirahmadi, Mohsen Noorinezhad

**Affiliations:** 1 Department of Environmental Health Engineering, Faculty of Health, Bushehr University of Medical Sciences, Bushehr, Iran; 2 The Persian Gulf Marine Biotechnology Research Center, The Persian Gulf Biomedical Sciences Research Institute, Bushehr University of Medical Sciences, Boostan 19 Alley, Imam Khomeini Street, Bushehr, Iran; 3 Systems Environmental Health, Oil, Gas and Energy Research Center, The Persian Gulf Biomedical Sciences Research Institute, Bushehr University of Medical Sciences, Bushehr, Iran; 4 The Persian Gulf Tropical Medicine Research Center, The Persian Gulf Biomedical Sciences Research Institute, Bushehr University of Medical Sciences, Bushehr, Iran; 5 The Persian Gulf Studies and Researches Center Marine Biotechnology Department, Persian Gulf University, Bushehr, Iran; 6 School of Public Health, Tehran University of Medical Sciences, Tehran, Iran; 7 Ecology Department, Iranian Shrimp Research Institute, Bushehr, Iran

**Keywords:** Aquatic Organisms, Environmental Monitoring, Geologic Sediments, Metals, Persian Gulf, *Trachycardium lacunosum*, Toxicology

## Abstract

**Background:**

The present work was designed to detect heavy metal contents of Al, Zn, Fe, Mn, Ni, V, Co, Cr and Cu in sediments and shells of the *Trachycardium lacunosum* collected in polluted and unpolluted areas along the Persian Gulf.

**Methods:**

The samples were taken from surface sediments (0-10 cm) and shells of *Trachycardium lacunosum* in two separated areas (polluted and unpolluted) in northern part of the Persian Gulf, Asaluyeh Bay, during summer 2013. The prepared samples were analyzed by inductively coupled plasma-optical emission spectrometry (ICP-OES).

**Results:**

Based on the results, all measured metals including Al, Zn, Fe, Mn, Ni, V, Co, Cr and Cu were meaningfully higher in the sediment samples of polluted area compared to unpolluted area and the order of metal concentrations in the sediment samples were Cr > Co > V > Ni > Zn > Cu > Fe > Al > Mn in polluted area. In the case of shell samples of *Trachycardium lacunosum*, polluted area contained significantly higher contents of Al, Zn, Fe, Mn, Ni, Co, Cr and Cu compared to unpolluted area and the order of metal concentrations in the shell samples were Fe > Zn > Al > Mn > Cu > Cr > Ni > Co in the polluted area.

**Conclusion:**

It was concluded that shells of the *Trachycardium lacunosum* can be used as a suitable bioindicator for heavy metals in the aquatic environment. Results confirmed that due to the possible contaminations by oil and gas activities near the polluted area perennial monitoring and mitigation measures is extremely necessary.

## Background

Environment protection needs awareness of the circumstance of the environments and the way in which they change. Hence, deterioration due to human and industrial activities and change in environments are the principal topics of monitoring studies [1]. The data attained in monitoring studies may use as a basic for managers and policy makers for evaluation and enhancement of environment condition by imposing proper actions to protect the environment. Coastline areas are subject to suffer from different negative environmental impacts due to industrial and human activities. Chemical pollution associated with industrial production is the main concern in the marine environment [2]. Heavy metals are considered as one of the most critical contaminants in the marine environment due to their bioaccumulation and biomagnification throughout the trophic chain [3, 4]. Heavy metals toxicity in marine organisms, long residence time within trophic chains, as well as the probable risk of human exposure to heavy metals, makes it essential to evaluate the concentrations of them in the aquatic environment and organisms [5]. Heavy metals may also induce sublethal effect in marine organisms, such as disruption of homeostasis, and impairment at cellular and molecular levels [6]. Additionally, these impacts may seriously decrease the persistence capacity of the organism by enhancing susceptibility to diseases and impairment [7]. Sediments act as a reservoir for various pollutants such as heavy metals and while many bivalves existing inside sediment accumulate elevated concentration levels of metals with regard to their bioaccessibility [8]. The ecological significance of bivalves, their simplicity of applying, their vast distribution and numerous abundance, and their relative to polluted sediments make them suitable species for toxicity testing of sediment [9]. Metals accumulate differentially in the shells and soft tissues of bivalves [10] however there is no particular position on if the use of shells or soft tissues alone is preferred in evaluating of metal [11]. But soft tissues have received further consideration amongst researchists for metals monitoring mostly because of agreement with the US coastal mussel watch monitoring scheme [12]. However, shell can provide a more precise symptom of pollution and environmental change [13]; they give minor variation than the living organism’s tissue also present a historic record of metal level all over the organism’s life cycle. This record still preserved after organism death [14]. High levels of different metals in sediments and organisms of marine environment are a well-documented environment concern [15]. But there are a few comprehensive studies in the Persian Gulf region especially on evaluation of metal contents in the bivalve shells of *Trachycardium lacunosum* with its connection to metal contents in the sediments. *Trachycardium lacunosum* is a marine and infaunal bivalve as well as a filter feeder pelecypod that belongs to the Cardiidae family. This bivalve has a white-rimmed shell, with the characteristic pink, brown, and purple spots overt. The average *Trachycardium lacunosum* length is about 25–35 mm. *Trachycardium lacunosum* is native to intertidal zone and sandy substrates of the Persian Gulf [16]. Due to the high dispersion of this bivalve in Nayband Bay and Lavar-e-Saheli, in this study we used *Trachycardium lacunosum* to evaluate its efficiency as a suitable bioindicator for metals.

The Persian Gulf is one of the oldest sea passageways in the world, and nearly 45 % of natural gas and 57–66 % of known oil reserves of the world lie in the region of the Persian Gulf. The presence of large amounts of natural gas and oil has made the Persian Gulf as one of the most strategic waterway in the world. The Persian Gulf has been the main waterway for oil transport in the last decades and during our time has also suffered from repeated oil spills to its marine environment.

To the best of our knowledge there is no report on the concentrations of heavy metals in the shells of *Trachycardium lacunosum* also there is no detailed study on heavy metal contents in the northern part of the Persian Gulf. So in this study for the first time in the offshore South Pars, the northern part of the Persian Gulf, we aimed to (1) measure the contents of Al, Zn, Fe, Mn, Ni, V, Co, Cr, as well as Cu in the shells of *Trachycardium lacunosum* and sediments simultaneously in two separated areas (polluted and unpolluted) (2) comparison between the metal contents of sediments in the polluted and unpolluted areas as well as shells (3) determine the interrelationships between metal contents in the shells of Tracycardium lacunosum as well as the sediments in both polluted and unpolluted areas.

## Methods

### Study area description

**Fig. 1:**
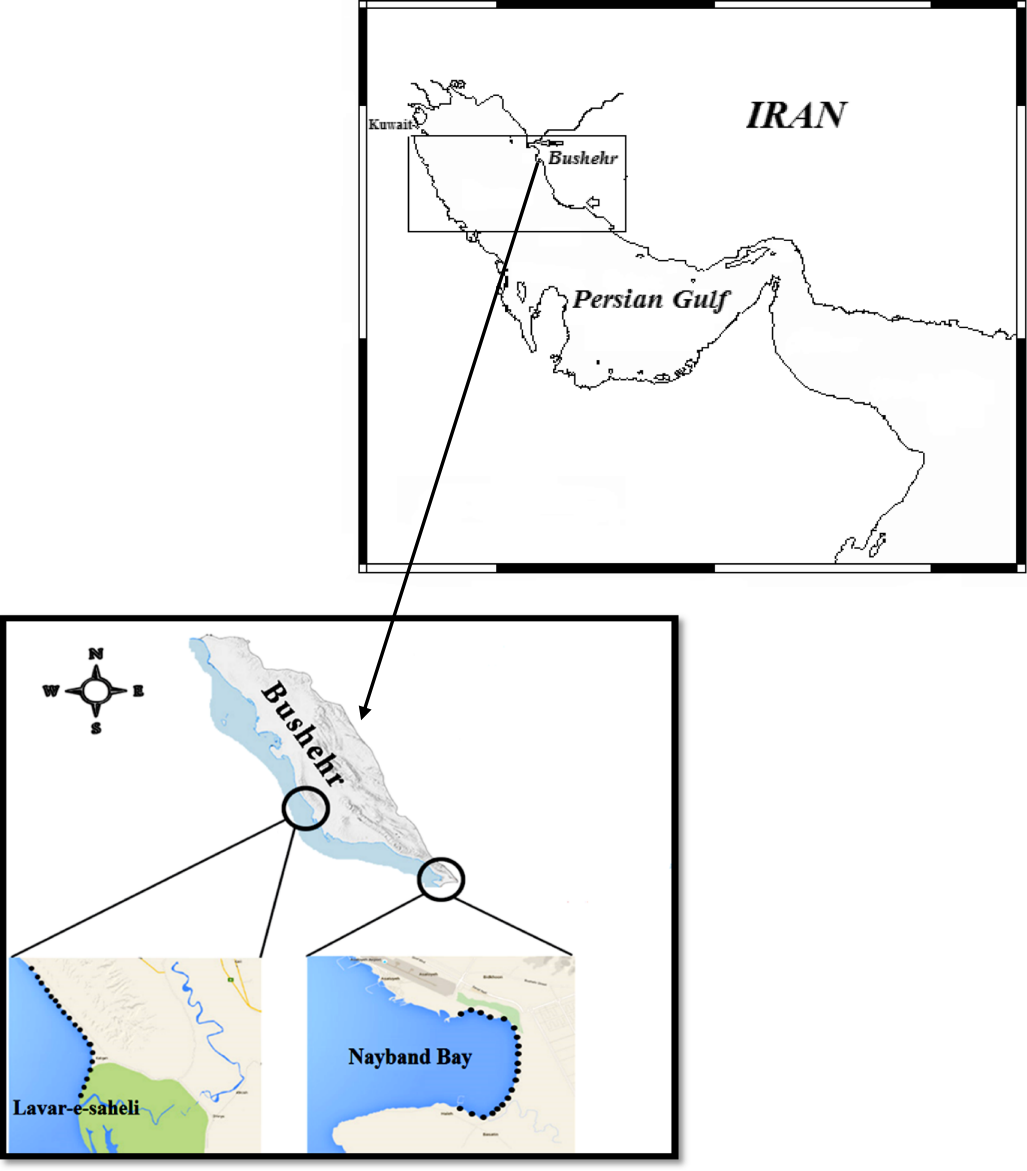
The map and locations of sampling stations in the study areas

The South Pars/North Dome is the world’s biggest gas field, shared between Iran and Qatar, and situated in the Persian Gulf. This natural gas field covers a space of 9700 km^2^ and the name of this field in Iranian territorial is South Pars. Closest land point to this gas field in the northern part of the Persian Gulf is Asaluyeh. It was chosen as the site for all facilities related to this gas field in Iranian territorial. Asaluyeh is situated on the shore of the Persian Gulf in southeast of Bushehr province. Two different areas were selected in the Asaluyeh as sampling points including polluted area (Nayband Bay) and unpolluted area (Lavar-e-Saheli) (Fig. 1 and Table 1). The surface sediment textures of both polluted and unpolluted areas are silt-clay.

**Table 1:** Geographical coordinates of the stations studied

Stations of unpolluted area	Number	E
1	28°13'45.59"N	51°17'12.51"E
2	28°13'42.77"N	51°17'13.12"E
3	28°13'40.38"N	51°17'13.37"E
4	28°13'38.14"N	51°17'13.72"E
5	28°13'36.13"N	51°17'13.94"E
6	28°13'34.10"N	51°17'14.12"E
7	28°13'33.77"N	51°17'14.45"E
8	28°13'32.27"N	51°17'14.67"E
9	28°13'30.01"N	51°17'15.02"E
10	28°13'27.49"N	51°17'15.31"E
11	28°13'24.97"N	51°17'15.76"E
12	28°13'22.36"N	51°17'16.18"E
13	28°13'19.04"N	51°17'16.46"E
14	28°13'16.31"N	51°17'17.74"E
15	28°13'14.09"N	51°17'17.81"E
16	28°13'12.08"N	51°17'17.68"E
17	28°13'5.35" N	51°17'17.71"E
18	28°13'2.74" N	51°17'17.86"E
19	28°12'59.71"N	51°17'17.64"E
Stations of polluted area	Number	E
20	27°26'39.57"N	52°40'32.36"E
21	27°26'21.06"N	52°40'34.43"E
22	27°26'2.91" N	52°40'36.37"E
23	27°25'48.69"N	52°40'35.29"E
24	27°25'33.86"N	52°40'35.21"E
25	27°25'21.54"N	52°40'33.93"E
26	27°25'11.54"N	52°40'32.18"E
27	27°25'1.77" N	52°40'29.43"E
28	27°24'52.85"N	52°40'25.78"E
29	27°24'45.36"N	52°40'22.32"E
30	27°24'36.78"N	52°40'18.48"E
31	27°24'27.10"N	52°40'14.73"E
32	27°24'19.29"N	52°40'9.73" E
33	27°24'11.30"N	52°40'5.49" E
34	27°24'4.09" N	52°40'0.34" E
35	27°23'57.01"N	52°39'52.23"E
36	27°23'50.64"N	52°39'41.26"E
37	27°23'49.45"N	52°39'4.93" E
38	27°23'46.16"N	52°39'15.78"E
39	27°23'43.78"N	52°39'27.46"E

### Sample collection

Composite samplings based on area (3 different locations for each sample) were performed at low tide times from the tidal area along the Persian Gulf coastal. Samples were collected from surface sediments (0-10 cm) and shells of *Trachycardium lacunosum* in both polluted and unpolluted areas during summer 2013 as fallow:

- In polluted area: 20 sediment samples and 18 shell samples
- In unpolluted area: 19 sediment samples and 13 shell samples

After transporting the collected surface sediments to the laboratory, the samples were dried at 105 °C for 24 h, homogenized, and packed in polyethylene bags and kept at -20 °C before analysis. The shell samples washed under a jet of water to liminate algae, sand, clay as well as other impurities, and then dried at 105 °C for 24 h and kept at -20 °C before analysis.

### Reagents

All the employed oxidants and mineral acids (HNO_3_, H_2_O_2_, HF, and HCl) were of suprapure quality (Merck, Germany). All plastic and glassware were cleaned by drenching overnight in a 10 % (w/v) HNO_3_ solution and afterward washed with deionized water before use. All solutions were prepared by ultrapure water (18.2 MΩ cm).

### Digestion and analytical procedures

The sediment samples (0.5 g) were digested with 6 ml hydrochloric acid (37 %), 2 ml nitric acid (65 %) in a microwave digestion system for 30 min and then diluted to 25 ml with ultrapure water and stored in polyethylene bottle until analysis. 0.5 g of powdered shell was fully digested in a Teflon cup using a mixture of conc. HNO_3_, HClO_4_ and HF with the ratio 3:2:1 respectively. Acids were added to dried sample and left overnight prior to further process. After that the samples were heated at 200°C then left to cool and filtered. The filtered solution was justified to a volume of 25 ml. It should be noted here that shell samples with similar shell length were selected for analysis in each sample point to minimize effects of body weight [17]. The bivalve length was measured by using a caliper with an accuracy of 0.02 mm. Blank digest was similarly performed. Metals analysis of Al, Zn, Fe, Mn, Ni, V, Co, Cr as well as Cu was performed by inductively coupled plasma optical spectrometry (ICP-OES). In Table 2, specifications of the instrumental operating circumstances are shown. All metal levels were represented as μg g^−1^ dry wet (dw). Statistical analysis of data was performed with the SPSS, Version 21 and Mann-Whitney U test as well as the Spearman’s rho correlation coefficient were used for statistical significant differences. Differences in mean values were accepted as being significant if *P* < 0.05.

**Table 2:** ICP-OES instrumental operating details

Parameters	
Company, model	SPECTRO (Germany), Spectro arcos
RF generator power (W)	1400
Frequency of RF generator (MHz)	27.12 MHz
Type of detector	Charge coupled devices (CCD)
Torch type	Flared-end EOP torch 2.5 mm
Plasma, auxiliary, and nebulizer gas	High purity (99.99 %) argon
Plasma gas flow rate (l/min)	14.5
Auxiliary gas flow rate (l/min)	0.9
Nebulizer gas flow rate (l/min)	0.85
Sample uptake time (s)	240 total
Delay time of (s)	-
Rinse time of (s)	45
Initial stabilization time (s)	Preflush: 45
Time between replicate analysis (s)	-
Measurement replicate	3
Pump rate	30 RPM
Element (λ/nm)	Al 396.152; Cu 324.754; Fe 259.941 Mn 257.611; Ni 231.604; Zn 268.416 Cr 205.618; Co 228.616; V 292.402

## Result and discussion

### Content of metals in sediments and shells

The concentration levels of examined metals (Al, Zn, Fe, Mn, Ni, V, Co, Cr and Cu) in sediment samples of polluted (Nayband Bay) and unpolluted (Lavar-e-Saheli) areas are shown in Table 3.

**Table 3:** Concentration of heavy metals (μg g^−1^ dw) in sediment samples at polluted & unpolluted areas

Area	Station	Al	Zn	Fe	Mn	Ni	V	Co	Cr	Cu
	1	0.136	5.4	0.162	0.006	2.1	6.1	16	1.5	2.2
	2	0.160	5.7	0.176	0.007	2	4.4	25	7.3	4.3
	3	0.201	6	0.273	0.010	3.1	8.8	22	64.9	1.2
	4	0.208	5.2	0.224	0.009	4.2	7.1	23	1.3	2.5
	5	0.251	6.2	0.278	0.010	3.7	7	22	3.5	2
	6	0.336	6	0.302	0.012	2.8	9.8	22	12.6	0.9
	7	0.526	6.1	0.411	0.018	6.4	4.4	22	70.7	0.1
	8	0.704	7.9	0.482	0.024	7.8	6.4	25	1.5	2.6
	9	0.811	9.2	0.461	0.023	9.4	6.1	22	58.6	2.3
Unpolluted area	10	0.799	8.7	0.445	0.023	8.1	13.4	23	74.7	3.8
	11	0.466	6.4	0.340	0.016	2.1	10.1	24	2.4	3.7
	12	0.109	3.2	0.095	0.005	2.2	7	25	1.5	1.9
	13	0.141	4.4	0.150	0.006	2.6	5.4	23	1	4.5
	14	0.074	4.7	0.065	0.004	2.2	4.1	25	1.5	3.3
	15	0.171	7.3	0.129	0.005	4.7	6.8	21	1.3	1.3
	16	0.145	4.6	0.169	0.007	2.1	4.1	24	6.3	2.1
	17	0.199	3.9	0.201	0.001	2.1	8	23	1.5	3.9
	18	0.144	3.8	0.170	0.007	2.2	2.8	23	1.3	2.7
	19	0.129	4.3	0.139	0.006	2.2	3.1	24	1.4	1.6
Mean ± SD		0.3005 ± 0.24	5.737 ± 1.65	0.246 ± 0.13	0.01 ± .006	3.79 ± 2.38	6.574 ± 2.66	22.85 ± 2.06	16.57 ± 27.1	2.47 ± 1.2
	20	0.161	5.4	0.171	0.007	5.9	5.6	29	170	1.9
	21	1.543	13.5	1.256	0.051	19.3	23.6	22	148.3	4.1
	22	1.488	15.6	1.532	0.053	19.4	27.2	26	438.3	1.6
	23	1.233	10.6	1.183	0.051	16	23.1	27	103.9	3.3
	24	1.108	10.3	1.150	0.049	17.4	21.7	21	93.8	8.3
	25	0.903	8.6	1.066	0.049	14.8	17	34	102.3	2.6
	26	0.928	10.1	1.067	0.048	16.9	21.6	35	92.3	1.8
	27	0.648	9.2	0.939	0.049	15.3	17.4	45	49.4	2.2
	28	0.831	9.6	0.997	0.048	15.3	13.3	24	48.9	4.2
Polluted area	29	0.988	11.1	1.188	0.050	16.4	19.1	34	71.5	3.8
	30	0.986	9.6	1.205	0.051	17	21.7	160	68.7	4.5
	31	1.167	11.5	1.243	0.049	18.4	26.4	21	96.1	2.5
	32	1.374	12	1.333	0.052	20.7	27	25	147.1	3.5
	33	0.843	9.9	1.144	0.048	16.4	13.4	25	53.6	2.9
	34	0.778	9.1	1.083	0.047	16.2	17.9	26	41.3	5.1
	35	0.712	11.2	1.016	0.049	13.6	19.4	35	44.9	2.7
	36	1.046	10.7	1.230	0.051	17.8	15.1	27	97	4
	37	0.955	11.2	1.211	0.054	14.8	23.5	22	76.6	3.1
	38	0.784	9.9	1.097	0.050	15.3	19.9	23	74.2	2.8
	39	0.739	8.2	1.051	0.048	16.2	13.7	25	65	2.7
Mean ± SD		0.960 ± 0.315	10.37 ± 2.05	1.108 ± 0.26	0.048 ± 0.009	15.490 ± 2.3	19.38 ± 5.4	34.3 ± 30.2	104.16 ± 86.4	3.38 ± 1.5

The orders of metal concentration levels in the sediment samples were Cr > Co > V > Ni > Zn > Cu > Fe > Al > Mn in the polluted area (Nayband Bay) and Co > Cr > V > Zn > Ni > Cu > Al > Fe > Mn in the unpolluted area (Lavar-e-Saheli).

In the unpolluted area the contents of Al, Zn, Fe, Mn, Ni, V, Co, Cr and Cu ranged from 0.074–0.811 (Mean: 0.3005), 3.2–9.2 (Mean: 5.737), 0.065–0.482 (Mean: 0.246), 0.004–0.024 (Mean: 0.01), 2–9.4 (Mean: 3.79), 2.8–13.4 (Mean: 6.57), 16-25 (Mean: 22.85), 1–74.7 (Mean: 16.57), and 0.1–4.5 (Mean: 2.47) μg g^−1^ respectively. In the polluted area the contents of Al, Zn, Fe, Mn, Ni, V, Co, Cr as well as Cu in the sediment samples ranged from 0.161–1.543 (Mean: 0.960), 5.4–15.6 (Mean: 10.37), 0.171–1.532 (Mean: 1.108), 0.007–0.054 (Mean: 0.048), 5.9-20.7 (Mean: 15.490), 5.6–27.2 (Mean: 19.38), 21–160 (Mean: 34.3), 41.3–438.3 (Mean:104.16), and 1.6–8.3 (Mean: 3.38) μg g^−1^ respectively. Ismail and Safahieh measured the content levels of Cu and Zn in the sediment samples collected from intertidal areas in the Lukut River. They have reported that Cu and Zn in the surface sediments were within the range of 37 to 100 μg g^−1^ and 100 to 210 μg g^−1^ respectively [18]. According to Usero et al. report, the concentrations of Cr, Cu, Pb, Zn, As and Hg in the sediments of Atlantic coast in southern Spain ranged from 10–33, 3–13, 0.26–0.72, 2–46, 18–460, 3.5–102 and 0.11-0.41 mg kg^−1^ dry mass respectively [19]. In another study, Palpandi and Kesavan measured concentration levels of heavy metals including Zn, Mn, Cu, Al, Cr and Ni in the sediment samples of Velar estuary, Southeast coast of India. They reported that the mean concentration levels of Cu, Fe and Zn ranged from 39.28 ± 0.6, 178.28 ± 1.12, 16.28 ± 1.24, 542.00 ± 487.58, 9.44 ± 3.11 and 1.64 ± 1.20 μg g^−1^ respectively [20].

**Fig. 2:**
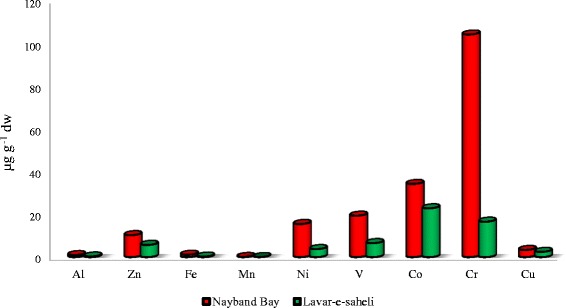
Comparison of heavy metal concentration levels in the sediment samples at polluted and unpolluted areas

Statistical analysis of Mann-Whitney U test showed that sediment samples in the polluted area contained significantly higher concentrations (*P* < 0.05) of all measured metals (Al, Zn, Fe, Mn, Ni, V, Co, Cr and Cu) compared to unpolluted area (Table 4). The comparison between metal concentrations in polluted and unpolluted areas are shown in Fig. 2.

**Table 4:** The differences between the metal concentrations of samples in polluted and unpolluted areas

Heavy metals	*P*-value sediments	*P*-value shells
Al	0.000	0.006
Co	0.009	0.000
Cr	0.000	0.000
Cu	0.021	0.001
Fe	0.000	0.000
Mn	0.000	0.000
Ni	0.000	0.009
V	0.000	-
Zn	0.000	0.000

Sediments act as both sinks and carriers for pollutants in the marine environments. Heavy metals are among the most usual marine contaminants and their occurrence in the marine environment indicates the presence of natural or anthropogenic source. Many studies have illustrated that heavy metal concentration in sediments can be sensitive indicators of pollutants in the marine environment [21, 22]. High concentration levels of trace metals in marine environments due to human activities have been recorded since old times. But elevated releases of toxic metals in to the municipal areas and the related health consequences just become clear in the 1960s [23]. Our study showed higher contents of Al, Zn, Fe, Mn, Ni, V, Co, Cr, as well as Cu in the Nayband Bay (polluted area) compare to unpolluted area mainly due to the activities of all related industries to gas and oil field in the region, boat repairing platform, shipping activities and discharge of effluents from the domestic sources nearby. The activities of industries after a while can release a diversity of poisonous sand possibly poisonous contaminants into the environment [24]. In a recent study in Jade Bay in NW Germany, the trace metal pollution in surface sediment and suspended particulate substance was described. Various metals including As, Cd, Cu, Ni, Pb, Sn and Zn were increased in the surface sediments. The potential metal sources in the region were the harbor area, floodgates and dumped harbor sludge in different parts of the region [25]. In a study in the Montenegrin coastal area, the overall trend for the concentration levels of measured metals in sediment samples was Fe > Mn > Cr > Ni > Zn > Cu > Co. The result of this study showed the anthropogenic impacts on the metal concentration levels in the Montenegrin beach zone [26]. In another study at Vellar estuary, Southeast coast of India the order of metal accumulation was Fe > Al > Mg > Mn > Cd > Cu > Cr > Zn > Ni > Pb. It was reported that higher level of metals could be due to effluents from municipal, domestic and agricultural wastes [20]. The contents of Al, Zn, Fe, Mn, Ni, V, Co, Cr as well as Cu in the shell samples of *Trachycardium lacunosum* in polluted (Nayband Bay) and unpolluted (Lavar-e-Saheli) areas are given in Table 5.

**Table 5:** Concentration of heavy metals (μg g^−1^ dw) in the shell samples at polluted and unpolluted areas

Area	Station	Al	Zn	Fe	Mn	Ni	Co	Cr	Cu
	1	0.360	0.465	0.935	0.106	0.003	0.012	0.001	0.061
	2	0.409	0.528	0.809	0.155	0.005	0.012	0.001	0.043
	3	0.081	0.003	1.564	0.220	0.007	0.010	0.001	0.004
	4	0.586	0.756	0.526	0.070	0.020	0.015	0.001	0.043
	5	0.699	0.404	1.265	0.176	0.006	0.003	0.001	0.003
	12	0.682	0.274	1.307	0.242	0.005	0.000	0.001	0.008
Unpolluted area	13	0.516	0.666	1.102	0.140	0.002	0.015	0.001	0.003
	14	0.000	0.179	1.295	0.219	0.003	0.000	0.001	0.010
	15	0.257	0.332	0.942	0.176	0.003	0.004	0.001	0.009
	16	0.463	0.598	1.294	0.226	0.007	0.013	0.001	0.014
	17	0.180	0.232	0.694	0.179	0.003	0.015	0.001	0.003
	18	0.758	0.104	0.802	0.186	0.005	0.006	0.001	0.003
	19	0.314	0.405	0.838	0.199	0.003	0.013	0.001	0.003
Mean ± SD		0.408 ± 0.24	0.3805 ± 0.22	1.029 ± 0.3	0.176 ± 0.05	0.006 ± 0.004	0.009 ± 0.006	0.001 ± 0	0.016 ± 0.02
	20	0.260	0.335	0.645	0.234	0.003	0.013	0.001	0.003
	22	0.727	0.938	4.514	0.980	0.008	0.020	0.057	0.028
	23	0.624	0.805	2.415	0.743	0.005	0.015	0.019	0.049
	24	0.641	0.827	3.024	1.022	0.007	0.016	0.055	0.116
	25	0.213	0.880	1.623	0.299	0.021	0.013	0.118	0.303
	26	0.139	0.902	1.868	0.388	0.003	0.017	0.083	0.930
	27	0.678	0.977	3.208	0.285	0.227	0.019	0.071	0.484
	28	0.640	0.826	2.340	0.645	0.121	0.016	0.001	0.006
Polluted area	29	0.557	0.719	3.265	0.673	0.055	0.012	0.001	0.014
	31	0.608	0.784	1.998	0.550	0.020	0.015	0.146	0.046
	32	1.114	1.437	6.850	1.269	0.055	0.022	0.068	0.141
	33	1.249	1.611	1.911	0.264	0.076	0.014	0.104	0.600
	34	1.722	2.221	6.126	0.630	0.065	0.015	0.001	0.003
	35	5.360	6.915	1.828	0.416	0.003	0.013	0.013	0.003
	36	0.956	1.233	3.747	0.800	0.003	0.017	0.048	0.036
	37	1.335	1.722	2.333	0.375	0.225	0.017	0.157	0.339
	38	0.699	0.875	6.543	0.246	0.003	0.019	0.162	0.349
	39	0.383	0.901	2.790	0.350	0.234	0.015	0.242	1.677
Mean ± SD		0.995 ± 1.16	1.385 ± 1.45	3.170 ± 1.76	0.565 ± 0.3	0.063 ± 0.083	0.016 ± 0.002	0.075 ± 0.07	0.285 ± 0.43

**Fig. 3:**
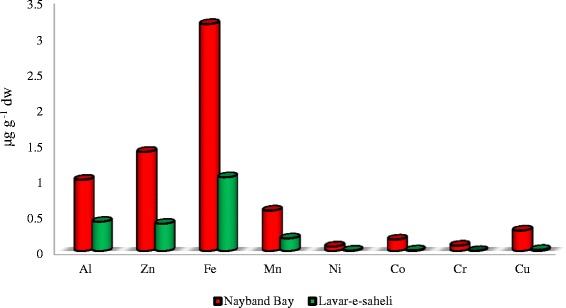
Comparison of heavy metal concentration levels in the shell samples at polluted and unpolluted area

The orders of metal concentration levels in shell samples were Fe > Zn > Al > Mn > Cu > Cr > Ni > Co in the polluted area (Nayband Bay) and Fe > Al > Zn > Mn > Cu > Co > Ni > Cr in the unpolluted area (Lavar-e-Saheli). In the polluted area the contents of Al, Zn, Fe, Mn, Ni, Co, Cr, and Cu in the shell samples ranged from 0.139–5.36 (Mean: 0.995), 0.335–6.915 (Mean: 1.385), 0.645–6.85 (Mean: 3.170), 0.234–1.269 (Mean: 0.565), 0.003–0.234 (Mean: 0.063), 0.012–0.022 (Mean: 0.016), 0.001–0.242 (Mean: 0.075), and 0.003-1.677 (Mean: 0.285) μg g^−1^ respectively. In the unpolluted area the concentration levels of Al, Zn, Fe, Mn, Ni, Co, Cr, and Cu ranged from 0–0.758 (Mean: 0.408), 0.003–0.756 (Mean: 0.3805), 0.526–1.564 (Mean: 1.029), 0.07–0. 242 (Mean: 0.176), 0.002–0.02 (Mean: 0.006), 0–0.15 (Mean: 0.009), 0.001–0.001 (Mean: 0.001), 0.003–0.061 (Mean: 0.016) μg g^−1^ respectively. In a study in Pantai Lido, west coast of Peninsular Malaysia, mean concentrations of Cu, Cd, Fe, Ni, Pb and Zn in the shell samples of *Perna viridis* were 8.41, 6.67, 48.3, 40.4, 59.4, and 5.96 μg g^−1^ respectively [27]. Ravera et al also determined the heavy metal levels in the shell samples of *Uniopictorium mancus* from shallow Bay located in Ranco, Italy. They reported that the mean values Al, Cu, Zn, Fe and Mn were found to be (80.86 ± 100.48), (3.53 ± 3.29), (24.00 ± 14.63), (211.20 ± 273.71) and (461.52 ± 252.67) μg g^−1^ respectively [28]. In a study in Tersakan River, south-west Turkey, mean concentration of Cd, Co, Cr, Cu, Fe, Mn, Ni, Pb and Zn in the shell samples of *Unio sp.* ranged from 0.382 ± 0.06, 1.155 ± 0.08, 7.403 ± 0.54, 15.902 ± 1.24, 671.182 ± 55.05, 268.291 ± 18.24, 20.821 ± 1.77, 4.157 ± 0.21 and 8.475 ± 2.48 μg g^−1^ respectively [29]. Statistical analysis of Mann-Whitney U test showed that Shell samples of *Trachycardium lacunosum* in polluted area contained significantly higher concentrations (*P* < 0.05) of all measured metals (Al, Zn, Fe, Mn, Ni, Co, Cr and Cu) compared with unpolluted area (Table 4). The comparison between metal concentrations in the polluted and unpolluted areas are shown in Fig. 3.

Beside sediment that may be good indicators of long and medium term of metal loads, bivalve shell is also an indicator of metal contamination since it is sessile and sedentary and reflects the metal level of the special region [30]. In the marine environments, metals discharged from sewage or industrial effluents may be quickly transported from water column to the sediment [31]. The accessibility of various metals in sediments provides a chance for marine organisms to biomagnify these metal and later remobilized them via the food chain. The metal concentrations in the shell samples of *Trachycardium lacunosum* in polluted area were higher than those of the samples taken from the unpolluted area. This indicated that the polluted area had higher pollution and bioaccessibilities of heavy metals. These results are in accordance with the fact that there are different anthropogenic activities, such as petrochemical plants and harbor activities in the Nayband Bay. Use of bivalve shells for metal contamination monitoring in the aquatic environments has various advantages over that of soft tissues. The shells are simple to keep and handle and become clear to be sensitive to environmental metals over the long period. As shells growth occurs incrementally they can provide an indication over a distinct time period, unlike the soft tissues which are good accumulator of various metals and integrate the chemical pollution indication over the living of the marine organisms [32].

The findings of this study showed that *Trachycardium lacunosum* is a good biological indicator for all examined metals except V in the Persian Gulf coastal areas due to its capability in bioaccumulating of metals from the sediment. In a study, Palpandi and Kesavan measured the levels of metals in sediment, shell and soft tissues of mangrove gastropod Nerita Crepidularia. They have reported that the order of metal accumulation in shell and soft tissues of Nerita Crepidularia was Fe > Al > Mg > Mn > Cd > Cu > Cr > Zn > Ni > Pb. They concluded that the higher levels of metals could be due to the heavy inflow of freshwater, which brought lot of effluents from municipal drainage and irrigation channels [20]. In another study it has been reported that between measured metals, Zn had the highest concentration level in the shell samples of *Perna viridis* and *Modiolus metcalfei* in Vellar Estuary, South East shoreline of India [33]. In another study in the Egyptian Red Sea shoreline, significant spatial differences in the metal concentration levels in *Tridacna maxima* were observed. The concentrations of most investigated metals in the *Tridacna maxima* shells and sediments were higher in the anthropogenic areas compare with unpolluted areas [34].

### Identification of metal interrelationships

The Spearman’s rho correlation coefficients were calculated to assess the association of metals in the sediment (Table 6) and shell samples (Table 7) in polluted and unpolluted areas.

**Table 6:** The Spearman’s rho correlations between metal concentrations in the sediments in polluted and unpolluted areas

		Al	Co	Cr	Cu	Fe	Mn	Ni	V	Zn
Unpolluted area	Al	1.000	–0.306	0.505^b^	–0.069	0.947^a^	0.928^a^	0.632^a^	0.558^b^	0.813^a^
	Co		1.000	0.015	0.358	–0.196	–0.163	–0.230	–0.269	–0.338
	Cr			1.000	–0.293	0.549^b^	0.505^b^	0.309	0.255	0.486^b^
	Cu				1.000	0.009	0.038	–0.323	–0.011	–0.124
	Fe					1.000	0.989^a^	0.513^b^	0.474^b^	0.710^a^
	Mn						1.000	0.486^b^	0.444^b^	0.654^a^
	Ni							1.000	0.221	0.616^a^
	V								1.000	0.432
	Zn									1.000
polluted area	Al	1.000	–0.328	0.867^a^	0.173	0.886^a^	0.689^a^	0.757^a^	0.722^a^	0.728^a^
	Co		1.000	–0.246	–0.232	–0.343	0.018	–0.238	–0.256	–0.304
	Cr			1.000	–0.154	0.735^a^	0.632^a^	0.585^a^	0.656^a^	0.594^a^
	Cu				1.000	0.141	0.203	0.143	–0.105	–0.081
	Fe					1.000	0.767^a^	0.759^a^	0.740^a^	0.765^a^
	Mn						1.000	0.411	0.477^a^	0.606^a^
	Ni							1.000	0.510^b^	0.517^b^
	V								1.000	0.751^a^
	Zn									1.000

^a^Correlation is significant at the 0.01 level

^b^Correlation is significant at the 0.05 level

**Table 7:** The Spearman’s rho correlations between metal concentrations in the shells in polluted and unpolluted areas

		Al	Co	Cr	Cu	Fe	Mn	Ni	Zn
Unpolluted area	Al	1.000	–0.042	.	–0.092	–0.130	–0.187	0.515	0.371
	Co		1.000	.	–0.142	–0.604^b^	–0.376	–0.034	0.578^b^
	Cr			.	.	.	.	.	.
	Cu				1.000	0.098	–0.313	0.312	0.352
	Fe					1.000	0.420	0.232	–0.301
	Mn						1.000	–0.092	–0.534^b^
	Ni							1.000	0.268
	Zn								1.000
polluted area	Al	1.000	0.158	–0.224	–0.352	0.282	0.083	0.016	0.779^a^
	Co		1.000	0.304	0.276	0.551^b^	0.191	–0.062	0.129
	Cr			1.000	0.809^a^	–0.184	–0.602^b^	0.161	0.012
	Cu				1.000	–0.170	–0.567^b^	0.230	0.006
	Fe					1.000	0.400	0.021	0.017
	Mn						1.000	–0.248	–0 .150
	Ni							1.000	0.101
	Zn								1.000

^a^Correlation is significant at the 0.01 level

^b^Correlation is significant at the 0.05 level

As shown in Table 4, most metals in the sediment samples in the polluted area are well correlated. Fe had remarkable positive correlations (*P* < 0.01) with Mn (*r* = 0.767), Ni (*r* = 0.759), V (*r* = 0.740), and Zn (*r* = 0.765). Cr had also noticeable correlations (*P* < 0.01) with Fe (*r* = 0.735), Mn (*r* = 0.632), Ni (*r* = 0.585), V (*r* = 0.656), and Zn (*r* = 0.594). In the case of Mn remarkable positive correlations (*P* < 0.01) were observed vs V and Zn. The significant correlation between Al and other metals (except Cu, Co) in both polluted and unpolluted areas confirms that these metals are associated with alumina silicate minerals. Similar significant positive correlations between metals in the sediment samples have been reported in different areas [25, 35]. As seen in Table 4, in the shell samples of *Tracycardium lacunosum* in the polluted area there are correlations for Al vs Zn (*r* = 0.779), Cr vs Cu (*r* = 0.809) and in the cases of Co vs Fe (*r* = 0.557), Cr vs Mn (*r* = – 0.602), and Fe vs Mn (*r* = – 0.567). The correlations were significant at the level of 0.05 in the polluted area. The significant correlations found between heavy metals could be due to several reasons such as differences in the biological half-life and biochemical behaviors of metals found in the sediments and shells [36–38].

## Conclusion

In this work, the levels of metals including Al, Zn, Fe, Mn, Ni, V, Co, Cr and Cu were determined in the sediment and shell samples of the bivalve *Tracycardium lacunosum* from two areas (polluted and unpolluted) of Asaluyeh Bay, northern part of the Persian Gulf. This study was the first effort to consider shell of *Tracycardium lacunosum* as a bioandicator for monitoring of heavy metals. Results of this study indicated that all measured metals including Al, Zn, Fe, Mn, Ni, V, Co, Cr and Cu were significantly higher in the sediment samples of polluted area compared with unpolluted area. In the case of shell samples of *Trachycardium lacunosum,* polluted area contained significantly higher concentrations of Al, Zn, Fe, Mn, Ni, Co, Cr and Cu compared to unpolluted area. It was concluded that shells of the *Trachycardium lacunosum* can be applied as a suitable bioandicator for heavy metals in the marine environment. Results confirmed that due to the possible pollution by oil and gas activities near the polluted area continuing and permanent evaluating as well as mitigation measures in this area is highly necessary.

## Abbreviation

ICP-OES: Inductively Coupled Plasma-Optical Emission Spectrometry.
